# Astragaloside IV attenuates hypoxia/reoxygenation injury-induced apoptosis of type II alveolar epithelial cells through miR-21-5p

**DOI:** 10.1080/21655979.2021.1982845

**Published:** 2021-10-07

**Authors:** Hang Li, Chang Yao, Kaihu Shi, Yang Zhao, Jin Du, Dinghui Hu, Zuntao Liu

**Affiliations:** a Department of Cardiothoracic Surgery, Affiliated Hospital of Integrated Traditional Chinese and Western Medicine, Nanjing University of Chinese Medicine, Nanjing, Jiangsu Province, China; b Department of Cardiothoracic Surgery, Jiangsu Province Academy of Traditional Chinese Medicine, Nanjing, Jiangsu Province, China; c Department of Breast Surgery, Jiangsu Province Hospital of Chinese Medicine, Affiliated Hospital of Nanjing University of Chinese Medicine, Nanjing, Jiangsu Province, China

**Keywords:** Astragaloside IV, type II alveolar epithelial cell, hypoxia/reoxygenation injury, miR-21-5p, TLR4/NF-ΚB

## Abstract

We aimed to explore the role of miR-21-5p in the inhibitory effects of astragaloside IV (As-IV) on hypoxia/reoxygenation injury-induced apoptosis of type II alveolar epithelial cells. Rat type II alveolar epithelial cells RLE-6TN were cultured *in vitro* and randomly divided into control (C), hypoxia/reoxygenation injury (H/R), As-IV and miR-21-5p-siRNA + As-IV groups (n = 6). H/R model was established by 24 h of hypoxia and 4 h of reoxygenation. As-IV group was given 1 nmol/L As-IV and incubated for 1 h before modeling. MiR-21-5p-siRNA + As-IV group was transfected with 50 nmol/L miR-21-5p-siRNA. After 48 h, they were incubated with 1 nmol/L As-IV for 1 h before modeling. Cell viability was detected by cell counting kit-8 assay, and apoptosis rate was detected by flow cytometry. The expression levels of TLR4 and NF-κB were measured by immunofluorescence assay. The targeting relationship between miR-21-5p and TLR4 was determined by luciferase assay. Compared with H/R group, the cell viability, miR-21-5p, bax and cleaved caspase-3 expressions of As-IV group increased, apoptosis rate and Bcl-2 expression decreased, and TLR4 and NF-κB expressions were down-regulated (P < 0.05). Compared with As-IV group, the cell viability, miR-21-5p, bax and cleaved caspase-3 expressions of miR-21-5p-siRNA + As-IV group decreased, apoptosis rate and Bcl-2 expression increased, and the expressions of TLR4 and NF-κB were up-regulated (P < 0.05). As-IV up-regulates miR-21-5p expression, inhibits the TLR4/NF-κB signaling pathway and suppresses the apoptosis of type II alveolar epithelial cells during hypoxia/reoxygenation injury.

## Introduction

Hypoxia/reoxygenation (HR) in lung tissues leads to apoptosis of alveolar epithelial cells, pulmonary vascular endothelial cells and bronchial epithelial cells, which subsequently causes lung injury. Alveolar epithelial cells mainly consist of alveolar epithelial type I cells (ATI) and type II cells (ATII) [1]. ATII cells are a key structure of the distal lung epithelium where they exert innate immune response and work as progenitors of ATI cells, contributing to alveolar epithelial repair and regeneration. In healthy lungs, ATII cells coordinate the host defense mechanisms, not only generating a restrictive alveolar epithelial barrier but also orchestrating host defense mechanisms and secreting surfactant proteins, which play a crucial role in lung protection against pathogen exposure [2]. Normal functioning of ATII is important for maintaining the structure and functions of alveoli and local environmental homeostasis. Clinically, there are many diseases leading to HR of lung tissues. For instance, for patients undergoing lung cancer radical resection (lobectomy) in thoracic surgery, acute lung injury after surgery may occur after HR [3]. The apoptosis of ATII during HR causes disorders of structure, functions and internal environment of alveoli. Therefore, how to protect ATII during HR has attracted widespread attention [4]. As one of the major effective components contributing to the cardiovascular pharmacological activities of traditional Chinese medicine *Astragalus membranaceus*, astragaloside IV (As-IV) can protect lung tissues [5]. Microribonucleic acid (miRNA) is a single-stranded non-coding small RNA containing approximately 21–23 nucleotides, with highly conserved sequences [6]. It specifically identifies the corresponding target site in the 3ʹ untranslated region (3ʹ UTR) of the target gene at the post-transcriptional level and regulates protein synthesis by inhibiting or degrading specific target genes [7]. With the discovery of miRNAs, the research on diseases has entered the post-gene era. MiR-21-5p is able to relieve cardiomyocyte apoptosis after rat myocardial overexpression during early ischemia reperfusion injury (IRI) [8]. Moreover, miR-21-5p is highly expressed in ATII and significantly down-regulated in the process of cell apoptosis. Up-regulating the expression level of miR-21-5p in cells can mitigate lung injury and reduce ATII apoptosis [9]. Therefore, rat ATII RLE-6TN cells were herein used to prepare the HR injury model, and then small molecule interference and As-IV alone or in combination were performed for intervention, aiming to evaluate the protective effects of As-IV on type II alveolar epithelial cells against HR injury and to clarify the mechanism.

## Materials and methods

### Cells

Rat ATII RLE-6TN cells (ATCC, USA) were inoculated into a culture plate and cultured with Dulbecco’s Modified Eagle Medium (DMEM) (Gibco, USA) containing 10% fetal bovine serum in an incubator at 37°C with 5% CO_2_.

### Main reagents and apparatus

As-IV was purchased from Nanjing Spring & Autumn Biotech Engineering Co., Ltd. (purity: one spot on a developed TLC plate, HPLC ≥98%, molecular formula: C41H68O14, molecular weight: 784.98) and DMSO was dissolved as the storage solution at a final concentration of ≤0.1%. Anti-Toll-like receptor 4 (TLR4), nuclear factor-kappa B (NF-κB), p-AMPK, p-mTOR, Beclin 1, LC3-II and p62 antibodies were sourced from Abcam, and horseradish peroxidase-labeled goat anti-rabbit secondary antibody was purchased from Shanghai Boyun Biotech Co., Ltd. Primer sequences were designed and synthesized by Shanghai Generay Biotech Co., Ltd., and bicinchoninic acid (BCA) protein assay kit was provided by Beyotime Institute of Biotechnology. Cell counting kit-8 (CCK-8) was bought from Dojindo, and reverse transcription kit was sourced from Thermo Fisher. The other reagents included high-glucose DMEM, fetal bovine serum and trypsin. Normoxic incubator and microrefrigerated centrifuge were sourced from Thermo Fisher, and superclean bench was purchased from Suzhou Antai Airtech Co., Ltd. Multifunctional microplate reader, GStorm Gradient PCR and protein electrophoresis/transmembrane apparatus were supplied by Bio-Rad.

### Cell grouping

The cells were divided into 4 groups using a random number table, i.e. control group (C group), HR injury group (H/R group), As-IV group and miR-21-5p-siRNA+As-IV group (n = 6). A cell HR injury model was constructed according to a previous literature [10]. The cells were cultured till they reached approximately 80% confluence. Then the medium was replaced by sugar- and serum-free DMEM. Later, the cells were cultured in a hypoxic incubator (1% O_2_, 5% CO_2_, and 94% N_2_) for 24 h, followed by culture in a common incubator for 4 h. Subsequently, As-IV group was cultured with As-IV at a final concentration of 1 nmol/L for 1 h, and the HR injury model was constructed. MiR-21-5p-siRNA+As-IV group was transfected with 50 nmol/L miR-21-5p-siRNA, followed by incubation with 1 nmol/L As-IV for 1 h and construction of the HR injury model. The primer sequences of miR-21-5p-siRNA were as follows: forward: 5ʹ-3ʹCGAGCUGCAAGAUUAACGAdTdT-3ʹ, reverse: 5ʹUCGUUUCUUGCGCUCGdTdT-3ʹ. Plasmid transfection was carried out in accordance with the instructions of Lipofectamine 3000 assay kit (Thermo Fisher, USA).

### Detection of cell proliferation by CCK-8 assay

In line with the steps prescribed in the kit’s instructions, the cells were inoculated into a 96-well plate at a density of 1 × 10^5^/well. Model replication was conducted after the cells attached to the wall. CCK-8 mixture (CCK-8:DMEM = 1:10) was prepared, 110 μL of which was added to each well containing the specimen. Then, the 96-well plate was placed into a normoxic incubator for incubation of 0.5–2 h. Finally, the absorbance of each well was measured at 450 nm using a microplate reader [11].

### Determination of cell apoptosis through flow cytometry

Briefly, 5 × 10^5^ cells were centrifuged, washed with PBS and suspended by adding 500 μL of binding buffer. Then, 10 μL of Annexin V-FITC and PI were added and incubated for 30 min in dark. Finally, cell apoptosis was detected by flow cytometry and analyzed by NovoExpress software [12].

### Detection of protein expressions in cells through Western blotting

Cells were collected from each group and added into 1 mL of lysate mixture (PMSF:RIPA = 1:100) for lysis on ice for 30 min. The cells were ground properly using a cell scraper. Later, the cell suspension was collected and centrifuged, and the supernatant was collected after the suspension precipitated. After the protein concentration was measured by the BCA assay kit, protein was degenerated at a high temperature. Thereafter, SDS-PAGE and (wet) transmembrane transfer were carried out, and the membrane was blocked with 10% skim milk for 2 h. After being washed with Tris-buffered saline-Tween 20 (TBST), the membrane was incubated with primary antibodies against Bax (1:200 diluted), Bcl-2 (1:500 diluted), cleaved caspase-3 (1:400 diluted), anti-TLR4 rabbit polyclonal antibody (1:100 diluted) and NF-κB mouse polyclonal antibody (1:100 diluted) (Abcam, USA) at 4°C overnight, followed by incubation with secondary antibody for 1 h on the next day. Then the membrane was washed with TBST 3 times, added with ECL chemiluminescent solution dropwise, and incubated for 2 min. Finally, exposure was conducted using a fluorescent chemiluminescent imaging device, and the grayscale value of the images was analyzed using Quantity One software [13].

### Detection of luciferase activity

Construction of mutant TLR4-UTR-luciferase reporter vector: Using wild-type STAT1-Mt luciferase reporter vector as the template, the miR-28 binding site on TLR4 3ʹ-UTR was subjected to site-directed mutagenesis using Q_uik Change Site-Directed Mutagenesis by PCR. Then the template plasmid was specifically removed by Dpn I enzyme, transformed and identified by sequencing. The mutant TLR4 3ʹ-UTR luciferase reporter vector was referred to as TLR4-Mut.

RLE-6TN cells were inoculated into a 24-well culture plate. When the cells reached 60–80% confluence, they were co-transfected with luciferase reporter vectors and miR-21-5p mimics, control mimics or miR-21-5p inhibitors, with p RL-TK transfection as the standard internal quality control. The cells were harvested at 36 h after transfection. The luciferase activity was detected according to the instructions of dual-luciferase reporter assay kit (Promega). Specifically, the medium was discarded, and the cells were washed twice with PBS. Then diluted l× PLB was added to each well for 15 min of lysis. Later, the cells were scraped off and placed into a 1.5 mL EP tube. After centrifugation at 12,000 r/min for 2 min, the supernatant was collected. Then 20 μL of cell supernatant was taken and added with 100 μL of human luciferase assay reagent (LAR II). The luciferase activity was detected with a single photon detector, and the firefly luciferase activity was recorded. After 10 μL of stop & Glo reagent was added, the Renilla luciferase activity was recorded using the single photon detector. Finally, the relative luciferase activity was calculated as follows: relative luciferase activity = firefly luciferase activity/Renilla luciferase activity [14].

### Statistical analysis

SPSS 26.0 software was employed for statistical analysis. All data were tested for homogeneity of variance and normal distribution. The normally distributed measurement data were expressed as mean ± standard deviation (mean ± SD) and examined by normality test. One-way ANOVA was applied for analyzing the means among multiple groups, while LSD-t test was used for pairwise comparison. The differences were statistically significant when P < 0.05.

## Results

### Cell viability

**Figure 1.**
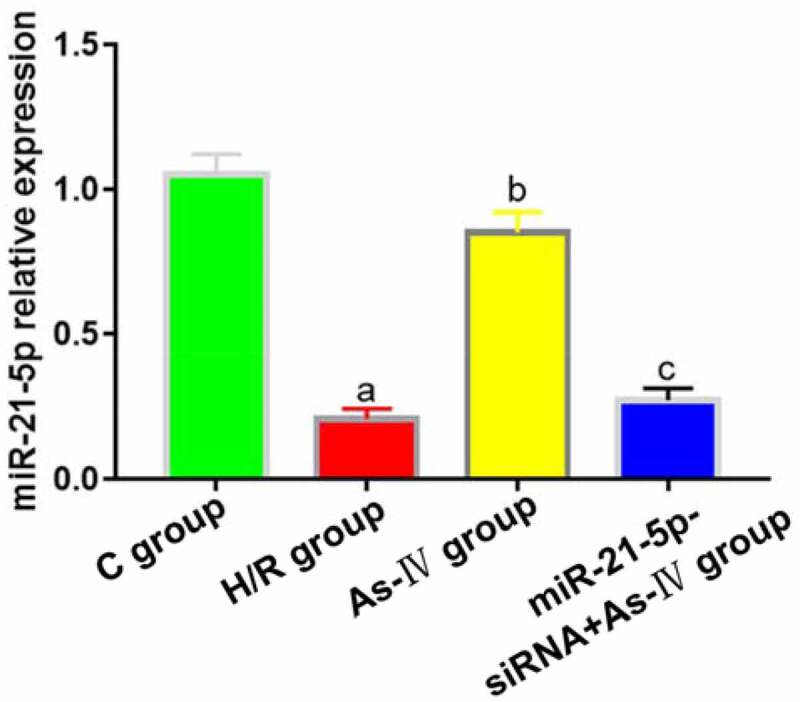
MiR-21-5p expression levels. ^a^*P* < 0.05 *vs*. C group, ^b^*P* < 0.05 *vs*. H/R group, ^c^*P* < 0.05 *vs*. As-IV group

As-IV can inhibit the proliferation and induce the apoptosis of renal cancer cells by regulating miR-21 gene [15], so miR-21 was selected as the target of As-IV in this study. Compared with C group, the expression of MiR-21-5p in H/R group significantly decreased (P = 0.013), and the expression level of MiR-21-5p in As-IV group was significantly higher than that in H/R group (P = 0.008). The expression levels of MiR-21-5p in MiR-21-5p-siRNA+As-IV and H/R groups were not statistically different (P = 0.412, 0.562) (Figure 1).

**Figure 2.**
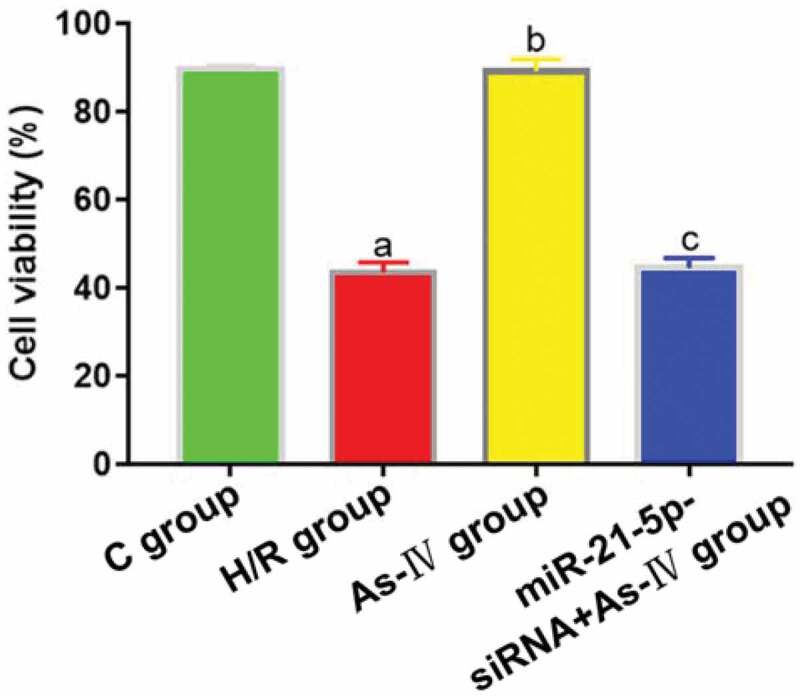
Cell viability. ^a^*P* < 0.05 *vs*. C group, ^b^*P* < 0.05 *vs*. H/R group, ^c^*P* < 0.05 *vs*. As-IV group

Compared with C group, the cell viability of H/R group decreased (P = 0.002), while that of As-IV group showed no significant change (P = 0.836). Compared with H/R group, the cell viability of As-IV group increased (P = 0.011), while that of MiR-21-5p-siRNA+As-IV group hardly changed (P = 0.845). Compared with As-IV group, the cell viability of MiR-21-5p-siRNA+As-IV group dropped (P = 0.002) (Figure 2).

### Cell apoptosis

**Figure 3.**
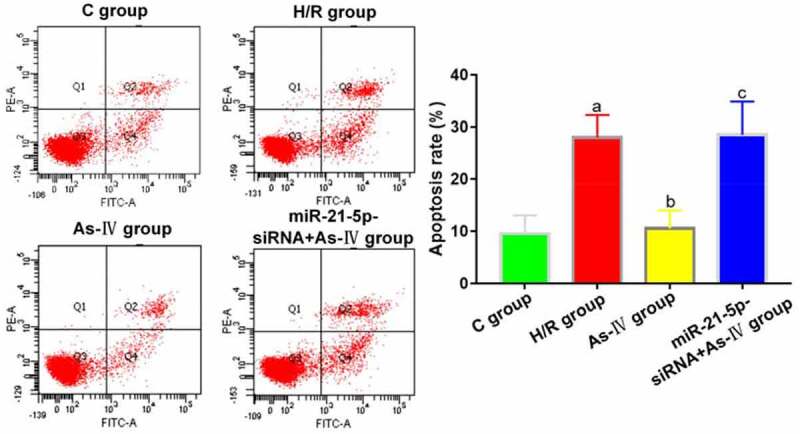
Cell apoptosis rates. ^a^*P* < 0.05 *vs*. C group, ^b^*P* < 0.05 *vs*. H/R group, ^c^*P* < 0.05 *vs*. As-IV group

Compared with C group, the cell apoptosis rate of H/R group significantly increased (P = 0.004), while that of As-IV group showed no significant change (P = 0.789). Compared with H/R group, the cell apoptosis rate of As-IV group significantly decreased (P = 0.024), while that of MiR-21-5p-siRNA+As-IV group barely changed (P = 0.614). Compared with As-IV group, the cell viability of MiR-21-5p-siRNA+As-IV group rose (P = 0.029) (Figure 3).

**Figure 4.**
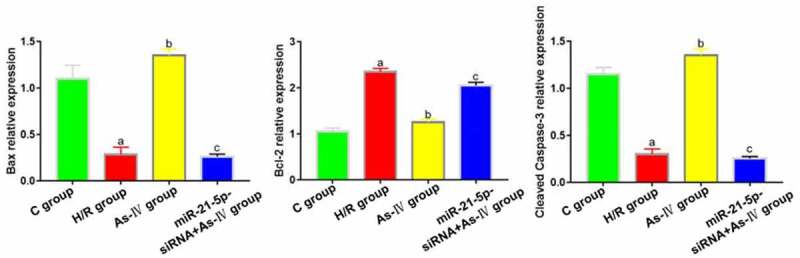
Expressions of apoptosis-related proteins Bcl-2, bax and cleaved caspase-3. ^a^*P* < 0.05 *vs*. C group, ^b^*P* < 0.05 *vs*. H/R group, ^c^*P* < 0.05 *vs*. As-IV group

Compared with group C, the expressions of bax and cleaved caspase-3 in H/R group decreased significantly (P = 0.017, 0.011) and that of Bcl-2 increased (P = 0.019). Compared with H/R group, the expressions of bax and cleaved caspase-3 in As-IV group significantly increased (P = 0.008, 0.006) and that of Bcl-2 decreased (P = 0.017). In miR-21-5p-siRNA+As-IV group, the expressions Bcl-2, cleaved caspase-3 and bax did not change significantly (P = 0.438, 0.539, 0,794). Compared with As-IV group, the expressions of bax and cleaved caspase-3 in miR-21-5p-siRNA+As-IV group significantly decreased (P = 0.028, 0.043) and that of Bcl-2 increased (P = 0.009) (Figure 4).

### TLR4 and NF-κB expressions

**Figure 5.**
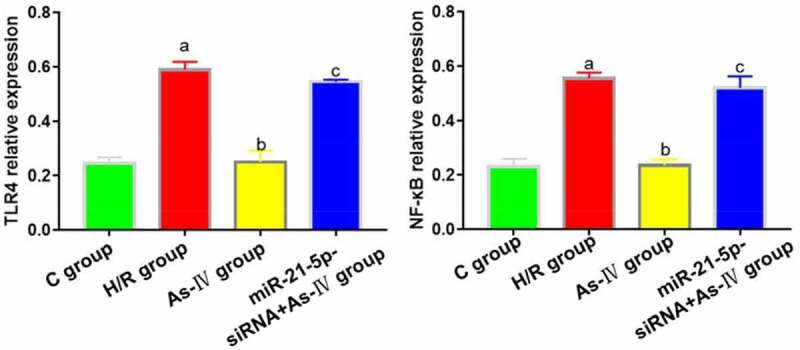
TLR4 and NF-κB protein expressions

7Compared with C group, the TLR4 and NF-κB expressions in H/R group increased (P = 0.004, 0.008), while those in As-IV group showed no significant changes (P = 0.873, 0.928). Compared with H/R group, the TLR4 and NF-κB expressions in As–IV group dropped (P = 0.032, 0.019), while those in MiR-21-5p-siRNA+As-IV group did not change significantly (P = 0.467, 0.889). Compared with As-IV group, the TLR4 and NF-κB expressions in MiR-21-5p-siRNA+As-IV group increased (P = 0.005, 0.018) (Figure 5).

### Targeting relationship between miR-21-5p and TLR4 determined by luciferase assay

**Figure 6.**
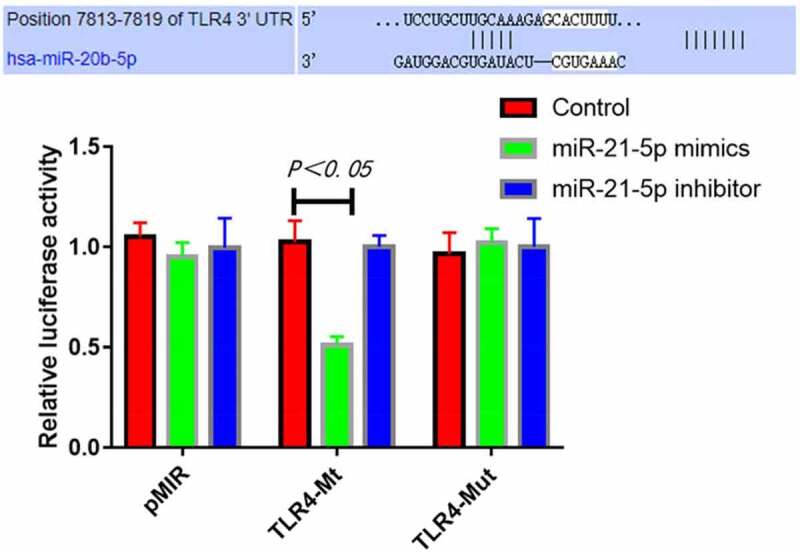
MiR-21-5p targeted and inhibited luciferase activity of TLR4

The results of luciferase assay revealed that compared with transfection with control mimics, transfection with miR-21-5p mimics significantly inhibited the luciferase activity of TLR4-Mt reporter vector (P = 0.019), while transfection with miR-21-5p inhibitor was unable to down-regulate the activity. In addition, miR-21-5p mimics had no obvious inhibitory effect on the luciferase activity of mutant TLR4-Mut reporter vector (Figure 6). Collectively, miR-21-5p can specifically bind 3ʹ-UTR of TLR4 mRNA.

## Discussion

ATII is one of the important cell populations on the alveolar surface, which secretes and synthesizes active substances on the lung surface, helps promote the transmembrane transport of fluid and electrolytes on the alveolar surface, supplements and differentiates ATI and plays an important role in detoxicating and metabolizing poisons and drugs. The apoptosis of ATII is involved in the entire process of acute lung injury [16]. In this study, the ATII HR injury model was established through 24-h hypoxia and 4-h reoxygenation to simulate IRI of the lungs. After HR, the viability of ATII decreased while the apoptosis rate increased, indicating successful construction of the ATII HR injury model. Additionally, compared with H/R group, the cell viability of As-IV group increased while the apoptosis rate reduced, suggesting that As-IV alleviated ATII HR injury.

The Toll-like receptor family comprises innate immune receptors widely distributed in lymphocytes, monocytes and macrophages. It plays a key role in innate immune defense by identifying ligand-pathogen-associated molecular patterns and subsequently mediating the release of inflammation mediators [17,18]. As a member of the Toll-like receptor family, TLR4 plays an important role in innate and adaptive immune responses. TLR4 participates in pulmonary IRI mainly by regulating NF-κB to activate downstream immune and inflammatory factors like TNF-α and IL-1 [19]. In this study, the expressions of TLR4 and NF-κB in ATII decreased after As-IV was administered, indicating that As-IV mitigated ATII HR injury by inhibiting the activation of the TLR4/NF-κB signaling pathway.

SiRNA technology has unique advantages such as high efficiency, specificity, stability, short cycle and simple operation. With reference to pre-experiment results, after miR-21-5p-siRNA at a final concentration of 50 nmol/L was applied to interfere with the expression of miR-21-5p in ATII, the pulmonary protection effect of As-IV was reversed and the expression levels of TLR4 and NF-κB also returned to the same levels in H/R group, indicating that As-IV can mitigate ATII HR injury by activating miR-21-5p and inhibiting the activation of the TLR4/NF-kB signaling pathway. In this study, it was also found by luciferase assay that miR-21-5p specifically bound 3ʹ-UTR of TLR4. This study provides a theoretical basis for the role of As-IV in the clinical treatment of alveolar structure and function disorders caused by apoptosis upon HR injury and pave the way for future drug research.

## Conclusion

In summary, the expression level of miR-21-5p in ATII decreases during HR injury, which rises after treatment with As-IV. Inhibiting the activation of TLR4/NF-κB signaling pathway attenuates cell apoptosis, and miR-21-5p can regulate TLR4 in a targeted manner.

## Highlights

- Astragaloside IV inhibits hypoxia-reoxygenation injury in alveolar epithelial cells.
- Astragaloside IV suppresses the activation of TLR4/NF-κB signaling pathway.
- The inhibition may be related to the activation of miR-21-5p.
